# How We Would Run a Journal

**Published:** 1901-11

**Authors:** 


### EDITORIALS.

### HOW WE WOULD RUN A JOURNAL.

Those who have never had the experience of running a journal to suit the other fellow who is on the outside have never known of half the vicissitudes of life. It is so easy to publish a journal, fill it full of good things, say nice things of this one and damn his neighbor, and pay for the privilege of so doing by going down in your pocket at the end of the year and making up the deficit, that we often wonder why we cling to such work and not let the other fellow, who knows ever so much better how to do it than we do, try it and pay for his experience. The newly-fledged graduate seems to think that we should spend all our efforts in jumping upon the non-graduate and crushing him at one full swoop, while many non-graduates write us of the failures of the college-bred men, and want the whole profession to know of their faulty judgment and lack of practical education. Then there is the fellow who cannot understand why we ever deign to recognize a non-graduate, especially one who, by force of some strong point of character, makes a place for himself and holds it. Then we must ever commend for public place and position only those who are college-bred and those of some special college, and condemn all other schools as insufficiently equipped to properly educate their students. Then there are those fellows who write us and tell us the author of some paper published, or the reporter of some case, does not know what he is talking about, and yet fails utterly to contribute anything that would shed any light upon the subject himself. Then there is another who thinks his paper should always be the leading article, and perhaps, on a look over the subscription-book, we find he has carried a subscription once in four or five years, or perhaps not at all. And another who feels that he ought to be on a free-list, and that the editors and publishers should deem it a special privilege to do all the work of keeping the journal going and, without a murmur, put up at the end of the year four or five hundred dollars to the printer for the purpose of allowing him to be free from a three-dollar subscription fee. We are open for proposals, under a sufficient guarantee, for some one to run this Journal and pay the deficit at the end of the year. We are praying and waiting for the Moses of veterinary journalism.

